# Therapeutic Potential of Ginsenosides as an Adjuvant Treatment for Diabetes

**DOI:** 10.3389/fphar.2018.00423

**Published:** 2018-05-01

**Authors:** Litao Bai, Jialiang Gao, Fan Wei, Jing Zhao, Danwei Wang, Junping Wei

**Affiliations:** Department of Endocrinology, Guang'anmen Hospital, China Academy of Chinese Medical Sciences, Beijing, China

**Keywords:** ginsenoside, herbal medicine, herbal active compounds, anti-diabetic effect, diabetes mellitus

## Abstract

Ginseng, one of the oldest traditional Chinese medicinal herbs, has been used widely in China and Asia for thousands of years. Ginsenosides extracted from ginseng, which is derived from the roots and rhizomes of *Panax ginseng* C. A. Meyer, have been used in China as an adjuvant in the treatment of diabetes mellitus. Owing to the technical complexity of ginsenoside production, the total ginsenosides are generally extracted. Accumulating evidence has shown that ginsenosides exert antidiabetic effects. *In vivo* and *in vitro* tests revealed the potential of ginsenoside Rg1, Rg3, Rg5, Rb1, Rb2, Rb3, compound K, Rk1, Re, ginseng total saponins, malonyl ginsenosides, Rd, Rh2, F2, protopanaxadiol (PPD) and protopanaxatriol (PPT)-type saponins to treat diabetes and its complications, including type 1 diabetes mellitus, type 2 diabetes mellitus, diabetic nephropathy, diabetic cognitive dysfunction, type 2 diabetes mellitus with fatty liver disease, diabetic cerebral infarction, diabetic cardiomyopathy, and diabetic erectile dysfunction. Many effects are attributed to ginsenosides, including gluconeogenesis reduction, improvement of insulin resistance, glucose transport, insulinotropic action, islet cell protection, hepatoprotective activity, anti-inflammatory effect, myocardial protection, lipid regulation, improvement of glucose tolerance, antioxidation, improvement of erectile dysfunction, regulation of gut flora metabolism, neuroprotection, anti-angiopathy, anti-neurotoxic effects, immunosuppression, and renoprotection effect. The molecular targets of these effects mainly contains GLUTs, SGLT1, GLP-1, FoxO1, TNF-α, IL-6, caspase-3, bcl-2, MDA, SOD, STAT5-PPAR gamma pathway, PI3K/Akt pathway, AMPK-JNK pathway, NF-κB pathway, and endoplasmic reticulum stress. Rg1, Rg3, Rb1, and compound K demonstrated the most promising therapeutic prospects as potential adjuvant medicines for the treatment of diabetes. This paper highlights the underlying pharmacological mechanisms of the anti-diabetic effects of ginsenosides.

## Introduction

**Figure 1:**
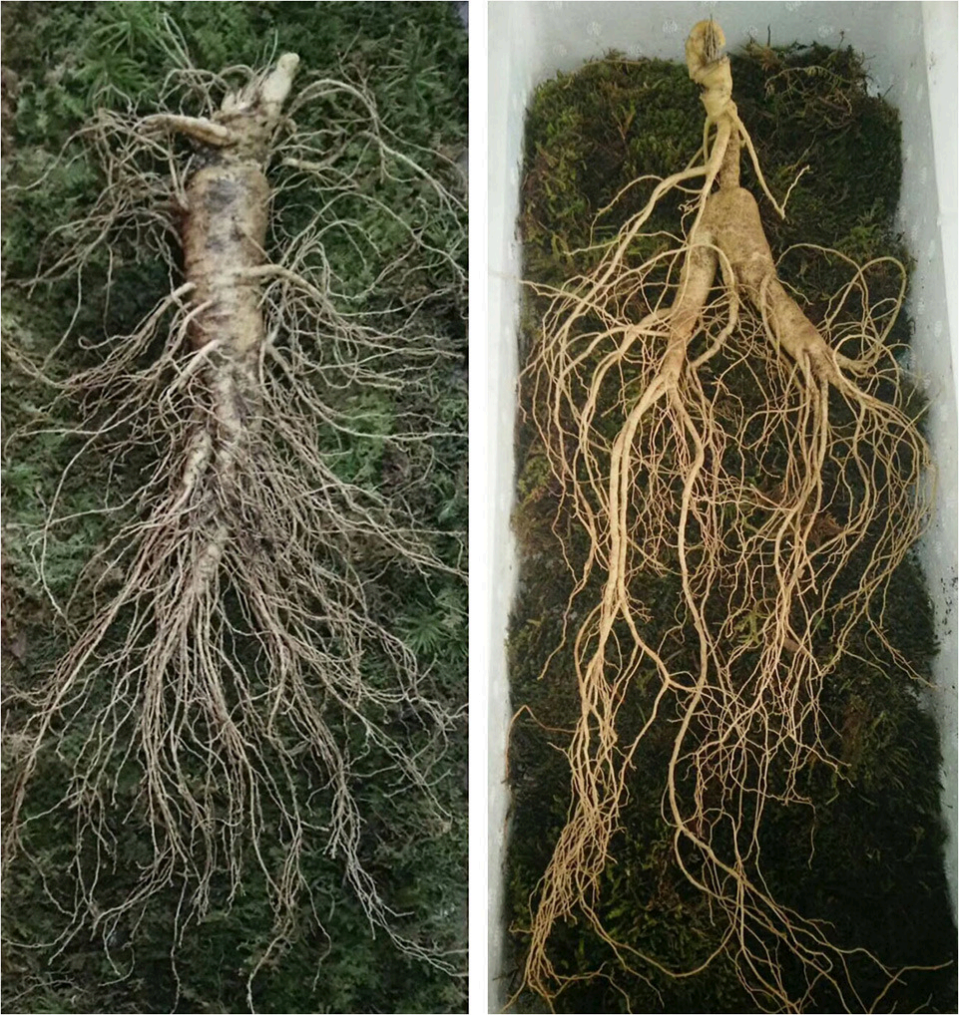
Root of Ginseng.

Ginseng, as a perennial herb of the genus Panax (Araliaceae family) with fleshy roots (Hu, 1977), has been widely used as a traditional Chinese Medicine for thousands years. The ginseng that is widely used as medical herbs includes *Panax ginseng* C. A. Mey (ginseng) and *Panax quinquefolium* L (American ginseng). Ginseng is mainly distributed in northeast China and Korean. Korean ginseng is shaped like ginseng in northeast China and almost has same effects. American ginseng is native both eastern American and Canada and is considered to have some similar efficacy and ingredients with ginseng. As one of the best-selling herbs in the world, ginseng and American ginseng are well documented in the China Pharmacopeia and the US Pharmacopeia, respectively. Ginseng (*Panax ginseng* C. A. Mey) has a wide range of their therapeutic functions anti-stress, health promotion, maintaining and enhancing central and immune systems, preventing certain chronic diseases, as well as aging deterrent properties. American ginseng seems to be more effective in cardiovascular disease treatment (Wang et al., 2015b) Traditional Chinese doctors believe that the feature of American ginseng (*P. quinquefolium* L) tends to be cold while ginseng (*Panax ginseng* C. A. Mey) tends to be warm. The present research focus on ginseng (*Panax ginseng* C. A. Mey), which is a perennial herb, with a root height of 30–60 cm, hypertrophic, fleshy, yellow-white, cylindrical or spindle-shaped, with a slightly-branched, short, upright rhizome (reed) (Figure 1). Currently, dry ginseng root is used worldwide to treat diabetes (Gui et al., 2016), cancer-related fatigue (Yennurajalingam et al., 2015), cardiovascular disease (Kim, 2012), stroke (Rastogi et al., 2014), and other diseases.

Ginsenoside is a triterpenoid saponin predominantly extracted from *P. ginseng* C. A. Meyer. Ginsenoside, the active ingredient in ginseng, is widely used in clinical practice as the main component of injections, granules, common tablets, dispersible tablets, capsules, and mixtures; thus, it has become a target of extensive research. The Japanese natural medicine chemist Shibata first identified the structures of various ginsenosides in 1965 (Shibata et al., 1965). Owing to the complexity of the extraction of ginsenoside monomers, most extractions of ginseng yield the total ginsenosides. At present, few manufacturers produce ginsenoside. This also limits the clinical use of ginsenosides.

Recent studies have shown that ginsenosides can be used to treat early chronic kidney disease (Xu et al., 2017), non-small-cell lung cancer (Leem et al., 2018), septic acute lung injury and acute respiratory distress syndrome (Sun et al., 2015). A randomized controlled trial showed that ginsenoside Rb1 ameliorates the renal function of patients with early chronic kidney disease. The trial recruited 197 patients with early chronic kidney disease and orally administered Rb1. Compared with that for the placebo group, renal function parameters (creatinine and urea clearance), oxidative stress, and inflammation significantly reduced (Xu et al., 2017). A meta-analysis comparing ginsenoside Rg3 combined with chemotherapy and chemotherapy alone for the treatment of non-small-cell lung cancer showed that the addition of ginsenoside Rg3 increased short-term efficacy, overall survival, and the proportion of CD4/CD8 T cells; these increases were statistically significant (Xu et al., 2016). Another clinical trial showed that ginsenosides acted synergistically with ulinastatin in the treatment of septic acute lung injury and acute respiratory distress syndrome. The pulmonary capillary permeability index, the extravascular lung water index, and the oxygenation index of the group treated with ginsenosides and ulinastatin group were significantly higher than those of the ulinastatin group. Hemodynamics and pulmonary circulation parameters, such as cardiac index, intracavitary blood volume, and central venous pressure, significantly improved (Sun et al., 2015). In addition, a wide range of pharmacological activities have been reported for ginsenosides, including anti-aging (Hu et al., 2015), immunoregulation (Yang et al., 2017), neuroregulation (Wang et al., 2016b), lipid regulation (Huang et al., 2017b), antithrombosis (Ban et al., 2017), and wound healing (Li et al., 2017).

At present, owing to its effects on the endocrine system (Supplementary Table 1), ginsenosides have been widely used in the adjuvant treatment of diabetes and diabetic complications. A meta-analysis showed that ginseng reduced fasting blood glucose in patients. Ginseng also exerted antidiabetic effects as a supplemental treatment (Shishtar et al., 2014). Ginseng extracts significantly improved glucose tolerance, improved in plasma glucose and insulin levels. In addition, it has antioxidant, anti-inflammatory, anti-apoptotic and immune-stimulatory activities (Jia et al., 2009). Many studies have reported the antidiabetic activity of ginsenosides. Ginsenosides may improve blood glucose through the regulation of glucose absorption (Shang et al., 2014), intervention in glucose transport and/or glucose disposal (Wang et al., 2015a), and the alteration of insulin secretion and binding (Gu et al., 2013). As ginsenosides affect multiple metabolic pathways, their efficacy is complex; furthermore, the various ginsenoside monomer components are difficult to separate. The potential pharmacological mechanisms of ginsenosides are unclear.

## The history of ginseng

Fossilized ginseng dates back to the Tertiary period, approximately 60–70 million years ago. Ginseng is one of the most precious Chinese herbal medicines. In ancient China, ginseng was used for first aid, health care, and the treatment of coma, cardiovascular diseases, and gastrointestinal diseases. *Shen nong ben cao jing* is the earliest existing monograph of traditional Chinese medicine, from approximately 4,000 years ago, which chronicles the use of ginseng in China as a medicine to nourish the body and the proposal that ginseng can delay aging without delaying side effects (Sun et al., 2016). Diabetes was known as Xiaoke disease in ancient China. In the Han Dynasty, Zhang Zhongjing wrote a book called *Shang Han Za Bing Lun*, which stated that ginseng could be used to treat thirst as the primary symptom of Xiaoke disease. In the Song Dynasty, *Tai Ping Hui Min He Ji Ju Fang*, an official traditional Chinese medicine book, recorded the treatment of Xiaoke disease with ginseng. Many of the proprietary Chinese medicines approved by the Chinese government for the treatment of diabetes contain ginsenosides, such as Tianqi Capsule (Pang et al., 2017), Jinlida Granule (Tian et al., 2018), and ShenMai Injection (Zhang et al., 2008).

## Chemical constituents

**Figure 2:**
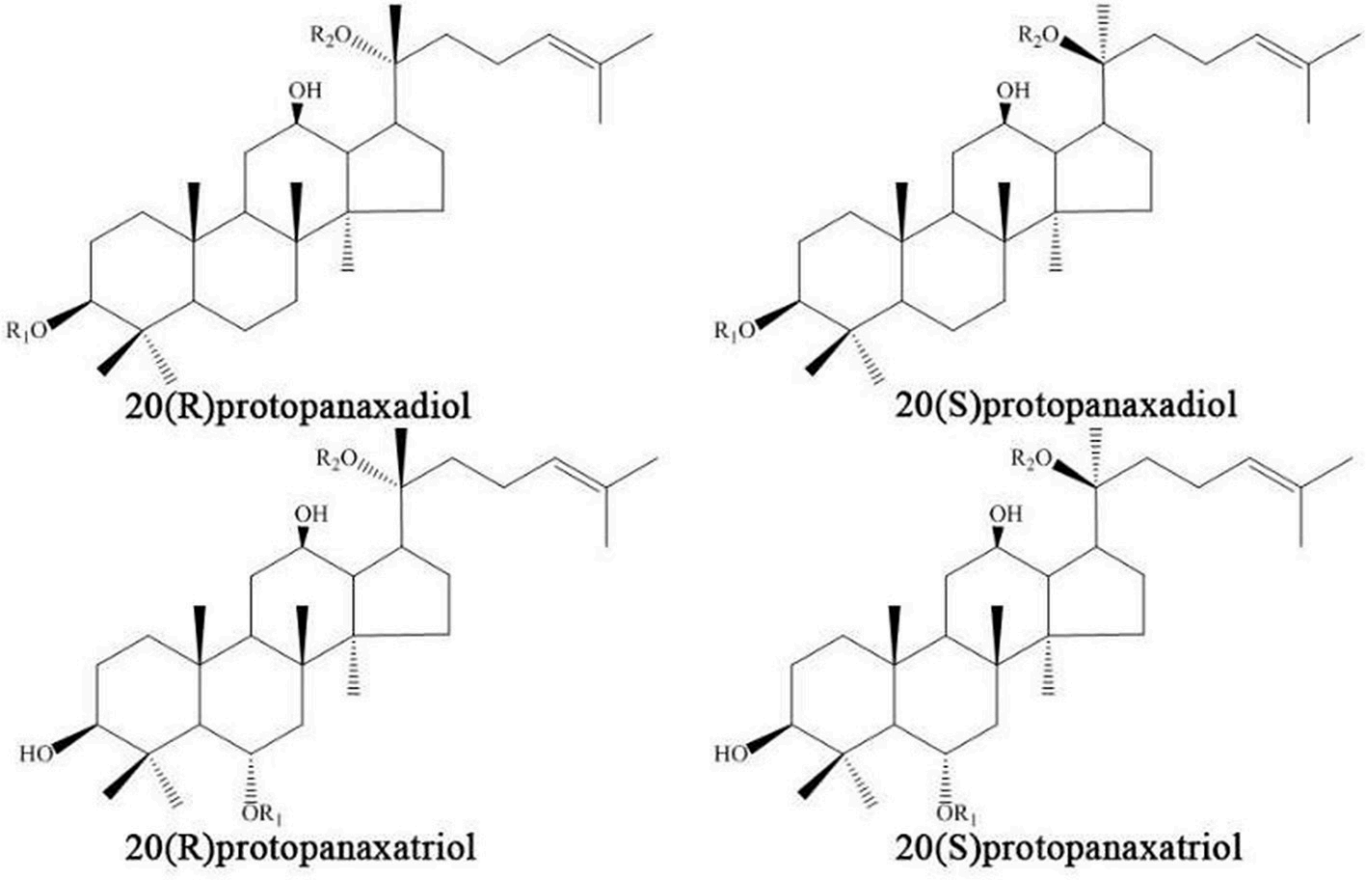
The protopanaxadiol (PPD) and protopanaxadiol (PPT) skeletons.

**Figure 3:**
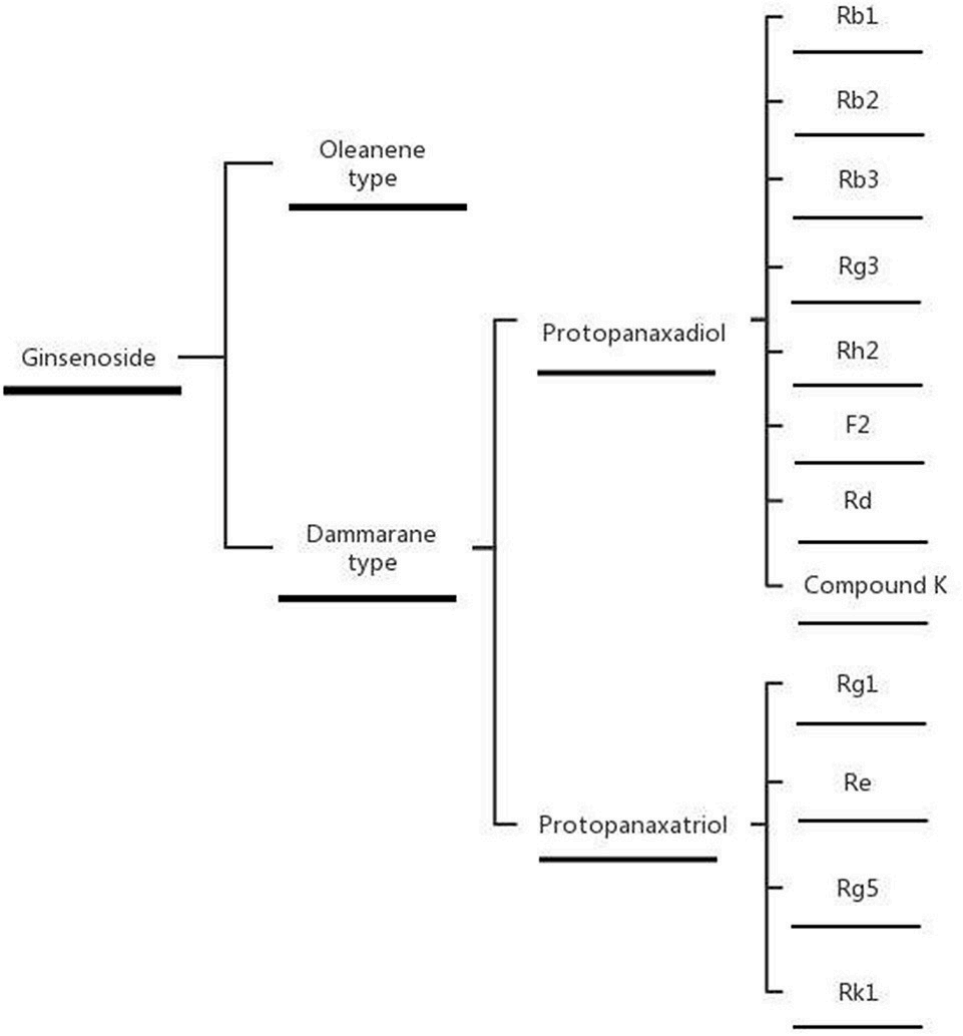
Classification of ginsenosides.

Depending on their structure, ginsenosides can be divided into dammarane type and oleanene type tricyclic triterpenoids. Dammarane type can be divided into protopanaxadiol and protopanaxatriol according to whether a C6-OH is present on the C-3, C-6, C-12, or C-20 of the skeleton. Furthermore, the C-20 of protopanaxadiol and protopanaxatriol is divided into 20(S) and 20(R)-type structures, depending on the position of the chiral carbon substitution (Leung and Wong, 2010). At present, 70 triterpenoid saponins have been isolated and identified from ginseng. In this study, we selected the ginsenosides with anti-diabetic effects, which can be divided into the following categories according to the structure of the skeleton (Figures 2, 3).

## Methodology

We searched PubMed for articles on ginsenoside-related diabetes from December 2012 to December 2017. The following search terms were used: Ginsenoside [All Fields] OR “ginsenosides” [MeSH Terms] AND (“diabetes mellitus” [MeSH Terms]) OR (“diabetes” [All Fields] AND “mellitus” [All Fields]) OR “diabetes mellitus” [All Fields] OR “diabetes” [All Fields] OR “diabetes insipidus” [MeSH Terms] OR (“diabetes” [All Fields] AND “insipidus” [All Fields]) OR “diabetes insipidus” [All Fields]) AND (“2012/12/10” [PDAT]: “2017/12/08” [PDAT]) AND (“2012/12/10” [PDat]: “2017/12/08” [PDat]). No language limitations were applied to the search.

## Inclusion criteria

The following inclusion criteria were defined: (a) the literatures are experimental articles; (b) the study medicine comprises ginsenosides extracted from ginseng (*Panax ginseng* C. A. Mey); (c) the articles study diabetes or diabetic complications.

## Exclusion criteria

The following inclusion criteria were defined: (a) excludes “ingredients identified” type articles; (b) the medicine contains other ingredients.

## Results

**Figure 4:**
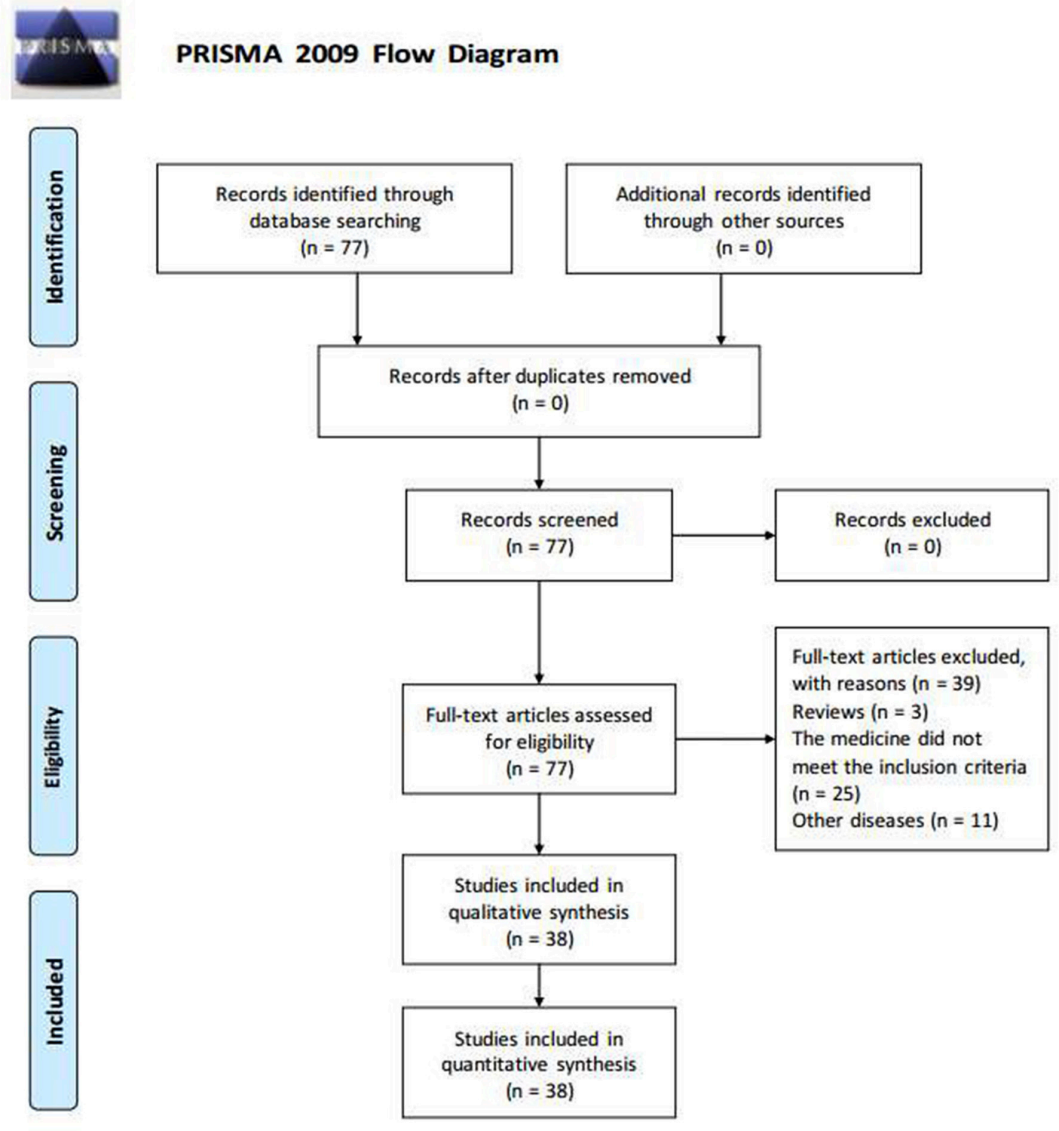
Flow Diagram.

The PRISMA flow diagram of article processing is shown (Figure 4): we screened 77 articles, from which the following were excluded: article type did not meet the inclusion criteria (three articles); study medicine is not ginsenoside extracted from the ginseng, or contains other Chinese ingredients (25 articles); the researched disease is not diabetes or diabetic complications (11 articles). After exclusion of the above 39 articles, we included 38 articles. Sixteen types of ginsenoside were found to have anti-diabetic effects, namely ginsenoside Rg1, Rg3, Rg5, Rb1, Rb2, Rb3, compound K, Rk1, Re, ginseng total saponins (GTS), malonyl-ginsenosides (MGR), Rd, Rh2, F2, and protopanaxadiol (PPD) and protopanaxatriol (PPT)-type saponins.

## Improving insulin resistance

Insulin resistance refers to a pathological condition in which the body cannot respond normally to insulin. Glucose intake and utilization decrease in the target organs, such as the liver, muscles, and adipose tissues, which releases excess glucose in the blood and results in increased blood glucose levels. At this point, the body is not sensitive to insulin, so there is a need for high levels of insulin to control blood glucose. At present, the main molecular mechanisms of insulin resistance include incorrect FFA regulation, abnormal adipogenic cytokines (Mlinar et al., 2007), insulin signaling disorders, and glucocorticoid excess. Glucocorticoid serves as an insulin antagonist that regulates insulin resistance and glucose intolerance. 11β-Hydroxysteroid dehydrogenase type 1 (11β-HSD1) enhances the function of glucocorticoid and thus triggers type 2 diabetes mellitus (T2DM) (Stimson et al., 2011; Akiyama et al., 2014). Ginsenoside Rb1 improved insulin resistance in high-fat diet (HFD)-induced T2DM mouse model through the inhibition of 11β-HSD1 to reduce blood glucose (Song et al., 2017). Endoplasmic reticulum stress is also a potential aggravating factor in insulin resistance (Flamment et al., 2012; Kim et al., 2017a). Previous studies have shown that in adipocytes, endoplasmic reticulum stress may lead to pathway dysfunction in insulin signaling, which causes adipocyte insulin resistance (Ozcan et al., 2004). Under conditions of endoplasmic reticulum stress, the ginsenoside complex Rk1 + Rg5 can improve insulin resistance and increase glucose uptake to provide protective effects in 3T3-L1 cells (Ponnuraj et al., 2014). Insulin resistance can also occur if any of the steps in the process of encoding the insulin gene into the glucose metabolism are altered. PI3K/Akt signaling pathway is an important signaling pathway involved in insulin resistance (Zhang et al., 2017). Downstream products of Akt/PI3K have a regulatory role in the utilization of glucose. The ginsenoside compound K, the final metabolic product of protopanaxadiol saponin, has an anti-diabetic effect (Shao et al., 2015). Compound K can improve insulin sensitivity through activation of the PI3K/Akt signaling pathway in diabetic rats (Jiang et al., 2014). Peroxisome proliferator-activated receptors (PPARs) are also key factors in the regulation of glucose and lipid metabolism in T2DM (Eldor et al., 2013). A clinical trial demonstrated that patients with T2DM had lower PPARgamma mRNA levels than normal controls (Ni et al., 2010). STAT5 triggers PPARgamma to modulate adipogenesis. *In vivo* and *in vitro* tests showed that Rg3 improved obesity-mediated insulin resistance, which was dependent on the downregulation of the STAT5-PPAR gamma pathway (Lee et al., 2017). The impairment of skeletal muscle glucose uptake also leads to insulin resistance. In skeletal muscle, mitochondrial dysfunction is a key factor of the insulin resistance (Di Meo et al., 2017). Evidence has shown that Rg3 activated mitochondrial functions, including the production of ATP and the consumption of oxygen, in C2C12 cells. Meanwhile, Rg3 improved the insulin signaling pathway and other related proteins. Rg3 ameliorates mitochondrial function, which has protective effects against insulin resistance in skeletal muscle (Kim et al., 2016). Malonyl ginsenosides (MGR) are a type of ginsenosides extracted from ginseng root that improve the effects of insulin resistance. In the glucose tolerance test, intraperitoneal injection of MGR (50 and 100 mg/kg/day, for 3 weeks) remarkably lowered fasting blood glucose (FBG) levels and increased glucose disposal. Meanwhile, MGR promoted insulin sensitivity in the insulin tolerance test (Liu et al., 2013b). In addition, brain insulin resistance is related to cognitive decline (Talbot et al., 2012). Patients with diabetics, especially those with T2DM, often perform poorly in learning and memory tasks. This is predominantly attributed to chronic hyperglycemia and microvascular disease (McCrimmon et al., 2012), called diabetic cognitive impairment. It is characterized by neural slowing, increased cortical atrophy, and microstructural abnormalities in white matter tracts. Research demonstrated that in adipocytes, c-Jun NH2-terminal kinase (JNK) caused the serine phosphorylation of insulin receptor substance-1, resulting in insulin resistance (Ozcan et al., 2004). *In vivo* experiments showed that ginsenoside Re improved cognitive behavior in mice with T2DM through increased acetylcholine (ACh) and inhibition of acetylcholinesterase (AChE) activity, and superoxide dismutase (SOD) and malondialdehyde (MDA) expression in brain tissues. The significant reduction of insulin resistance and hyperglycemia by ginsenoside Re may provide a potentially new strategy for the treatment of diabetes-related cognitive dysfunction. This may be associated with the ginsenoside-mediated reduction of oxidative stress and protection of the cholinergic neurons via the inhibition of the JNK pathway (Kim et al., 2017b).

## Improving glucose tolerance

Glucose tolerance refers to the ability of the body to regulate blood glucose levels. Impaired glucose tolerance increases the probability of progression to diabetes (Nathan et al., 2007). T2DM can be prevented by changes in lifestyle of subjects with impaired glucose tolerance (Tuomilehto et al., 2001). Therefore, the improvement of glucose tolerance is a key point in the prevention and treatment of diabetes. Ginsenoside Rb2 enhances autophagy through activation of the SIRT1 and AMP-activated protein kinase (AMPK) signaling pathways to reduce lipid accumulation caused by the combination of oleic acid and high glucose and therefore significantly improves glucose tolerance (Huang et al., 2017b). In addition, Rb1 (Song et al., 2017), compound K (Jiang et al., 2014), and protopanaxadiol (PPD) and protopanaxatriol (PPT)-type saponins (Deng et al., 2017) attenuated glucose tolerance to play a role in T2DM.

## Reducing gluconeogenesis

Gluconeogenesis, the increase in hepatic glucose production, is a vital element in the progress of glucose disorders (Bock et al., 2007). In physiological conditions, liver glycogen synthesis and gluconeogenesis remain in a dynamic equilibrium. However, when the liver appears insulin resistance, liver gluconeogenesis increased, whereas hepatic glycogen synthesis is reduced (Leclercq et al., 2007). After the balance of gluconeogenesis and glycogen synthesis is disrupted, liver glycogen output is increased, followed by the elevation of blood glucose. AMPK, as a key regulator of energy metabolism, reduces plasma, liver triglyceride levels and gluconeogenic gene transcription (Cool et al., 2006). Modern hypoglycemic medicines, such as metformin, inhibit gluconeogenesis through the activation of AMPK in liver cells to reduce blood glucose in type 2 diabetes (Zhou et al., 2001; Madiraju et al., 2014). Forkhead transcription factor 1 (FOXO1) is another important factor in gluconeogenesis. AMPK can regulate FOXO1, which suppresses hepatic gluconeogenesis (Zhang et al., 2009). Some ginsenosides exert blood glucose lowering effects in this way. Researches confirmed that ginsenoside Rg1 (Liu et al., 2017), Rb3 (Meng et al., 2017) and, compound K (Wei et al., 2015) reduced gluconeogenesis through increased AMPK expression and decreased FOXO1 activity, which may offer a potential treatment for type 2 diabetes. Ginsenoside Rg5 prevented gluconeogenesis via the suppressing of HIF-1α (Xiao et al., 2017) expression.

## Effects on glucose transport

Sugar transport is important for the regulation of blood glucose level (Chen et al., 2015). However, glucose cannot enter the cell freely through the lipid bilayer structure of the cell membrane. Glucose uptake by cells requires the help of glucose transporters to achieve transport function. Glucose transporters exist in various tissues of the body. They are divided into two groups: sodium-glucose cotransporters (SGLTs), which actively transport glucose against a concentration gradient; and the other, which facilitates the transport of glucose in a facile and diffusive manner along a concentration gradient without any energy expenditure during transport. Thus, the regulation of glucose transport and disposal are critical for the maintenance of blood glucose level. Ginsenoside Rb1 can promote the translocation of glucose transporter to increase glucose uptake in adipocytes. This reduced fasting glucose through a recovery in the expression of GLUT1 and GLUT4 and the phosphorylation of Akt in the adipose tissue of db/db mice (Shang et al., 2014). Glucose absorption is mainly mediated by transmembrane transport through the sodium-glucose cotransporters 1 (SGLT1). Studies have shown that intestinal SGLT1 levels were significantly increased in patients with diabetes (Dyer et al., 2002) and positively correlated with the pathogenesis of diabetes (Dominguez Rieg et al., 2016). A recent clinical study reported that the glycated hemoglobin levels of oral SGLT1 inhibitors were lower than placebo in patients with type 1 diabetes who were receiving insulin (Garg et al., 2017). Ginsenoside Rg1, through the regulation of SGLT1 gene expression to effectively reduce intestinal glucose uptake, provides a potential strategy for antihyperglycemia and antidiabetic treatments (Wang et al., 2015a). Ginsenoside Rg3, F2, compound K, and Rh2 can also inhibit SGLT1 (Gao et al., 2017a).

## Insulinotropic action

A characteristic of diabetes is that the pancreas cannot provide sufficient insulin or the body cannot respond properly to insulin. Thus, the increased secretion of insulin is a key prospect for the treatment of T2DM. Glucagon-like peptide-1 (GLP-1) has insulinotropic action (Nadkarni et al., 2014) and a β-cell protective function (Rondas et al., 2013); it acts on pancreatic β cells, promotes insulin synthesis and secretion, stimulates the proliferation and differentiation of pancreatic β cells, inhibits pancreatic β-cell apoptosis, and increases the number of islet β cells. Simultaneously, GLP-1 can also act on islet α cells to inhibit the release of glucagon. Thus, GLP-1 affects the maintenance of blood glucose homeostasis (Nadkarni et al., 2014). Rg3 stimulated GLP-1 secretion in enteroendocrine L cells and reduced hyperglycemia in a T2DM mice model through a sweet taste receptor-mediated signal transduction pathway (Kim et al., 2015). Thus, it is a potential medicine for T2DM and obesity. In an *in vivo* test, the administration of oral ginseng total saponins at 150 or 300 mg/kg per day for 4 weeks decreased fasting plasma glucose and postprandial plasma glucose, improved insulin secretion and lipid homeostasis, and ameliorated the HOMA-IR index. In an *in vitro* test, ginseng total saponins and ginsenoside Rb1 stimulated GLP1 secretion in cultured NCI-H716 cells to provide antidiabetic effects (Liu et al., 2013a). Thus, ginseng total saponins and Rb1 have long-term prospects in the fight against hyperglycemia and lipid metabolism disorders. In pancreatic beta cells, the secretion of insulin requires GLUT2 (Thorens, 2015). Compound K enhanced insulin secretion via the upregulation of GLUT2 in MIN6 pancreatic β-cells (Gu et al., 2013), and improved insulin levels and insulin resistance to combat T2DM.

## Protecting islet cells

Apoptosis is a form of β cell death that occurs in diabetes. AMPK is an important enzyme in the regulation of metabolism; it triggers the JNK switch directly to induce apoptosis. The pathological state of DM upregulates the expression of Bax/Bcl-2 and caspase-3, which contributes to islet cell apoptosis in mice with DM. Compound K decreases Bax/Bcl-2 and caspase-3 and protects pancreatic islet cells from apoptosis through the inhibition of the AMPK/JNK pathway and the subsequent suppression of the progression of T2DM (Guan et al., 2014). Moreover, islet transplantation, which benefits from the reduction of islet cell apoptosis, has recently emerged as a new method to control diabetes. In particular, it is a good alternative treatment for type 1 diabetes mellitus(T1DM) and has the advantages of safety and fewer adverse reactions than pancreas transplantation (Noguchi et al., 2006). Treatment with ginsenoside Rg3 before islet transplantation can increase islet cell function and reduce islet cell apoptosis. After Rg3 treatment, there was a significant improvement in total insulin release and pancreatic β cell apoptosis (Kim et al., 2014); thus, Rg3 confers protective effects on islet cells during islet transplantation.

## Regulation of gut flora metabolism

Gut flora is a complex microbial community in the digestive tract of humans, which is known to be related to the pathogenesis of diabetes. The characteristic of gut flora may be significantly altered in patients with diabetes. The regulation of the gut flora metabolism may decrease the influence of diabetes (Gao et al., 2017b). A study reported that 20(S)-ginsenoside Rg3 reduced blood glucose through the regulation of gut flora metabolism in rats with T2DM (Niu et al., 2012). Another study showed that Rb1 regulated the intestinal microflora to inhibit deglycosylation. Thus, it may exert a positive effect on the clinical management of diabetes (Liu et al., 2015a).

## Antioxidant effect

Oxidative stress is a pathological condition in which reactive oxygen species in the body lead to greater effects than an unbalanced redox reaction. Oxidative stress may play a key role in the pathogenesis and development of diabetes (Maritim et al., 2003). As the body produces excess reactive oxygen species, β-cell maturation and apoptosis increase and insulin synthesis and secretion decrease; diabetes, hyperglycemia, and obesity can increase the production of reactive oxygen species, which results in oxidative stress, creating a vicious circle in which oxidative stress and diabetes promote each other. Further studies have provided evidence that oxidative stress has a relationship with diabetic complications such as DN (Sagoo and Gnudi, 2018), diabetes with erectile dysfunction (Liu et al., 2015b), and diabetes with cognitive dysfunction (Kim et al., 2017b). The ginsenosides compound K (Shao et al., 2015), Rg3 (Liu et al., 2015b), and Re (Kim et al., 2017b) decreased the oxidative stress marker MDA, and enhanced SOD in animal models of Diabetic nephropathy(DN), diabetes with erectile dysfunction, and diabetes with cognitive dysfunction, respectively. Rg5 inhibits fatty acid oxidation against the hepatic glucagon response to reduce diabetes (Xiao et al., 2017). *P. quinquefolium* also has antioxidant effects. At the same time, it reduces NO level while there was no effect on C-peptide level (Amin et al., 2011). Ginsenoside Re could exert protective activity against the occurrence of oxidative stress in the eye and kidney of diabetic rats. It provides evidence that ginsenoside Re could be used to prevent diabetic microangiopathy (Cho et al., 2006a) 2006.

## Anti-inflammatory effects

Low-grade inflammation is a key cause of T2DM as it can lead to insulin resistance (Lackey and Olefsky, 2016). Pro-inflammatory macrophages may reduce the insulin sensitivity of the liver, skeletal muscle, and pancreatic β-cells. The suppression of the inflammatory response may represent a future therapy for T2DM. Rb2 upregulated GPR120 expression in RAW264.7 macrophages, which lowered the level of iNOS and COX-2 expression to provide an anti-inflammatory effect; thus, it may be a viable solution to relieve inflammation and improve glucose metabolism (Huang et al., 2017a). Intraperitoneal injection of Rb1 treatment also decreased the levels of pro-inflammatory cytokines, including TNF-α, IL-6 and or IL-1β and NF-κB pathway molecules (p-IKK and p-IκBα) in an animal experiment (Wu et al., 2014). PPD and PPT also reduced the expression of TNF-α and IL-6, which prevented T2DM (Deng et al., 2017). Many diabetic complications are also associated with inflammation. Ginsenosides can prevent diabetic complications through a reduction in the inflammatory response. A review confirmed that inflammation aggravated the progression of diabetic nephropathy. Ginsenoside 20(S)-Rg3 inhibited the inflammatory pathway to ameliorate this pathological condition (Kang et al., 2013). Rg3 reduced NO production and apoptosis to enhance islet function and ameliorate early inflammation after transplantation (Kim et al., 2014). Rg1 can also provide an anti-inflammatory effect by the inhibition of the JNK signaling pathway to prevent T2DM with fatty liver disease (Tian et al., 2017). In addition, protopanaxadiol saponin fraction decreased the release of inflammatory mediators such as nitric oxide (NO), tumor necrosis factor-α and prostaglandin E2 *in vitro* and *in vivo* inflammatory models (Yang et al., 2015). C-reactive protein is used mainly as a marker of inflammation, whose levels rise in response to inflammation. Ginsenoside Re regulated the level of C-reactive protein, indicating that Re might improve diabetes and its complications by relieving inflammation (Cho et al., 2006b).

## Lipid regulation

T2DM is closely linked to the epidemic of obesity (DeFronzo et al., 2015). It is particularly important for diabetic patients to actively prevent dyslipidemia. Lipid deposition in the liver causes liver insulin resistance (Park et al., 2016). Lipotoxicity increased islet cell apoptosis (Huang C. N. et al., 2017) and restricted the use of glucose capacity in muscle. Ginsenoside Rg1 can affect lipid metabolism in streptozotocin-induced type 2 diabetic rats. It inhibits the JNK signaling pathway to exert its anti-apoptotic and anti-inflammatory effects, and reduce the total cholesterol (TC), triglyceride (TG), and low-density lipoprotein cholesterol (LDL-C) (Tian et al., 2017). PPD and PPT-type saponins can reduce FBG and regulate serum lipid-related markers, such as reduced TC, TG, and LDL-C through the inhibition of the expression of liver metabolic genes in a high-fat diet and *Streptococcus*-induced type 2 diabetes mellitus (Deng et al., 2017). Ginsenoside Rg3 participates in the improvement of lipotoxicity. The lipid-regulating effect of Rg3 is dependent on the regulation of the STAT5-PPAR gamma pathway (Lee et al., 2017). The lipid-regulating effects of Rg3 also exerted beneficial effects against DN (Wang et al., 2016a). Moreover, Re (Kim et al., 2017b) and compound K (Jiang et al., 2014) also reduced the TG and TC to support lipid regulation.

## Immunosuppressive effect

T1DM also known as insulin-dependent diabetes, is a chronic condition in which the body produces insufficient insulin. It accounts for only approximately 5–10% of all cases of diabetes mellitus (DM). However, its incidence continues to increase all over the world and it has severe short-term and long-term implications (Daneman, 2006). The management of T1DM mainly comprises lifestyle interventions, insulin therapy, pancreas transplantation, and islet cell transplantation (Bruni et al., 2014). However, owing to the immune response, islet transplantation has a high failure rate (Campbell et al., 2007). Therefore, the exploration of new immunosuppressive medicine is necessary to increase the safety and effectiveness of islet transplantation. It was reported that compound K suppressed immune responses and prolonged transplanted islet survival in a mice model of T1DM; hence, it exerts potential therapeutic effects on islet transplantation (Ma et al., 2014).

## Hepatoprotective activity

Non-alcoholic fatty liver disease (NAFLD) is closely related to metabolic syndrome, especially when diabetes is involved. The liver is an important organ involved in glucose and lipid metabolism (Fracanzani et al., 2008). T2DM is an underlying factor in the occurrence of NAFLD. An animal experiment showed that ginsenoside Rg1 exerted hepatoprotective activity in a rat model of T2DM. Ginsenoside Rg1 decreased the blood glucose level and improved the insulin resistance index in rats with T2DM. Ginsenoside Rg1 also lowered the blood lipid profile, including TC, TG, and LDL-C levels and decreased aspartate transaminase and alanine transaminase levels. This hepatoprotective active is mainly mediated by anti-apoptotic effects, the suppression of JNK activity, and the inhibition of inflammation. The experiment reveals the clinical potential of Ginsenoside Rg1 as an adjuvant drug for the therapy of patients with T2DM and fatty liver disease (Tian et al., 2017). Ginsenoside Rb2 reduced hepatic lipid accumulation through the activation of the SIRT1 and AMPK signaling pathways, which induced autophagy to improve NAFLD (Huang et al., 2017b). In addition, animal experiments showed that compound K improved glucose intolerance and hepatic steatosis in T2DM OLETF rats, which a double effect that involves the reduced synthesis of fatty acids and the promotion of fatty acid oxidation (Hwang et al., 2017). These results suggest that compound K may have potential hepatoprotective functions.

## Diabetic cardiovascular complications

Cardiovascular complications are notable causes of death in diabetic patients (Bauters et al., 2003; Nathan et al., 2003). Diabetic cardiomyopathy is mainly manifested as myocardial dysfunction in the absence of other heart disease and may eventually progress to heart failure. Ginsenosides can provide myocardial protection through improved cardiac function, attenuated cardiac fibrosis, reduced myocardial apoptosis, and antioxidant activity. *In vivo* experiments showed that ginsenoside Rh2 improved heart function in a streptozotocin-induced model of type 1 diabetes in rats. *In vitro* experiments showed that Rh2 activated PPARδ in cardiomyocytes cultured in high glucose, which inhibited the expression of STAT3, reduced cardiac fibrosis, and protected against diabetic cardiomyopathy (Lo et al., 2017). Ginsenoside Rg1 decreased the percentage of apoptotic myocardial cells and increased the parameters of cardiac function; it prevented myocardial lesions and myocardial collagen volume fraction. In rat models of diabetes, the mechanism through which ginsenoside Rg1 ameliorates diabetic cardiomyopathy is the inhibition of ER stress-induced apoptosis (Yu et al., 2016). Another animal experiment showed that the treatment of ginsenoside Rg1 to diabetic rats was related to decrease oxidative stress and attenuated myocardial apoptosis. This indicated that ginsenoside Rg1 may be a potential compound to preventing cardiovascular impairment in diabetic patients (Yu et al., 2015).

## Improving endothelial dysfunction

Diabetes increases the risk of endothelial dysfunction (Ishida et al., 2014). A vitro experiment has shown that ginsenoside Re can improve the expression of endothelial cell function markers such as endothelin, nitric oxide, vascular endothelial growth factor and interleukin-6 (IL-6) during the early stage of diabetes. This effect of reducing endothelial dysfunction may be exerted by activating p38MAPK, ERK1 / 2 and JNK signaling (Shi et al., 2016). In human retinal endothelial cells, Rk1 regulated endothelial barrier function and markedly reduced the vessel leakiness of retina in a diabetic mouse model. This protective property of Rk1 might effectively control the endothelial leakage in diabetic retinopathy and other vascular leakage diseases (Maeng et al., 2013).

## Improving erectile dysfunction

Erectile dysfunction can be a complication of diabetes mellitus. Hyperglycemia damages the male reproductive functions, leading to erectile dysfunction, ejaculatory dysfunction, and decreases in semen volume, sperm count, and sperm motility (Maresch et al., 2018). Therefore, studies have assessed whether ginsenosides could reverse erectile dysfunction caused by hyperglycemia. It was deduced that the impairment of spontaneous erectile response in diabetic rats may be caused by the degeneration of neurons and oxidative stress. An *in vivo* test reported that the administration of Rg3 (100 mg/kg) by gavage enhanced erectile function in diabetic model rats. The anti-erectile dysfunction of Rg3 resulted from neuroprotective activity and antioxidant effects in corpus cavernosum cells (Liu et al., 2015b).

## Neuroprotective effect

Cerebral infarction is a diabetic cerebrovascular complication. Diabetes can lead to abnormal coagulation mechanisms, which result in an increased incidence of cerebral infarction in patients with diabetes (Putaala et al., 2011). Nerve cells in the ischemic state can be damaged, but ginsenosides offer some neuroprotective effects. An animal experiment demonstrated that ginsenoside Rg1 nanoparticles penetrated the blood-brain barrier, reduced the volume of diabetic rats with cerebral infarction, and promoted the recovery of neurons. Ginsenoside Rg1 nanoparticles are expected to provide a clinical treatment for cerebral infarction in DM (Shen et al., 2017).

## Anti-angiopathic effects

Diabetic microangiopathy and macrovascular complications, also known as diabetic angiopathy, are the leading causes of morbidity and mortality in DM. Diabetic retinopathy and DN are the most common diabetic angiopathies, and can result in great harm; thus, the study of therapeutic targets are of great importance. Ginsenoside Re has anti-angiopathy effects; it activates p38 MAPK, ERK1/2, and JNK signaling to prevent diabetic angiopathy in Wistar rats with DM (Shi et al., 2016).

## Anti-neurotoxic effect

In diabetic cognitive impairment, hyperglycemia may exert toxic impacts that result in brain function and structural abnormalities (Gispen and Biessels, 2000). Ginsenoside Rb1 displays anti-neurotoxic effects on neurons, which may be related to the regulation of endoplasmic reticulum stress. The involvement of endoplasmic reticulum stress is recognized in a variety of neurodegenerative diseases. Rb1 may protect neurons against high-glucose induced cell damage via the suppression of endoplasmic reticulum stress induced C/EBP homologous protein (CHOP), which may offer a novel strategy for the treatment of diabetic cognitive dysfunction (Liu et al., 2014). Furthermore, ginsenosides Rd and R-Rh2 prevented neurotoxicity in astrocytes. Ginsenosides Rd and R-Rh2 improved the cell viability of astrocytes, ameliorated insulin signaling and inhibited apoptosis. Thus, Rd and R-Rh2 may have therapeutic potential for the prevention of cognitive impairment caused by diabetes (Chu et al., 2014).

## Kidney protection effect

DN is one of the most important chronic micro-vascular complications of DM and has become the leading cause of end-stage renal disease worldwide (Gupta et al., 2011). DN increases the risk of premature death and presents a serious financial burden (Yang et al., 2010). Thus, the treatment of DN is topical, but complex, research. At present, the treatment of DN mainly includes the control of blood sugar and blood pressure levels, adherence to a low protein diet (Fried et al., 2013), and kidney replacement therapy. However, in the event of clinical DN, the kidney function continues to decline until the development of end-stage renal failure. Diabetes influences body's metabolism and blood circulation, which likely generates excess reactive oxygen species. These conditions injure the kidney's glomeruli and cause albuminuria (Cao and Cooper, 2011). As diabetic nephropathy progresses, the glomerular filtration barrier, which is composed of the fenestrated endothelium, the glomerular basement membrane, and the epithelial podocytes, becomes more damaged (Mora-Fernandez et al., 2014). Streptozotocin-induced DN rats displayed an aggravated volume of renal glomerulus, increased basement membrane, and higher mesenterium mass. At the same time, the renal glomerulus contains some inflammatory cells. Ginsenoside 20(S)-Rg3 is a key bioactive constituent of ginseng after heat-processing and is used for the treatment of pathological conditions associated with DN. It clearly suppressed inflammatory pathways via the inhibition of oxidative stress and advanced glycation end product formation. Meanwhile, Ginsenoside 20(S)-Rg(3) improved pathological conditions and renal damage in DN animal models (Kang et al., 2013). Compound K decreased renal function markers, blood urea nitrogen (BUN) and serum creatinine (Scr), in high-fat diet and streptozotocin-induced rats; it also improved renal tissue pathological changes, enhanced antioxidant effects, and reduced TGF-β1 in renal tissue damage to protect the kidney function of diabetic rats (Shao et al., 2015).

## Conclusion and perspective

The increase in the incidence of diabetes imposes serious social, financial, and health burden (Disease et al., 2016). According to the International Diabetes Federation (IDF), a total of 415 million adults worldwide have diabetes. China has the largest number of people with diabetes in the world, approximately 110 million (Ogurtsova et al., 2017); the overall incidence of diabetes in Chinese adults is 11.6% (Xu et al., 2013). DM increased the mortality and morbidity of patients. The treatment of DM mainly includes lifestyle interventions, drug treatment, and insulin treatment, but DM is a life-long chronic disease for which there is no cure. Traditional Chinese medicines (TCMs) have continued to make a significant contribution to the prevention and treatment of diabetes in China. Besides Routine treatment, the effect of TCMs is attributable to herbal ingredients containing hundreds of compounds and playing different roles. Through the administration of various compounds, TCMs achieve the goal of systematic treatment of complex diseases by affecting multiple targets. A number of double-blind, randomized, placebo-controlled, multicenter trials have shown that TCMs have a clear effect on diabetes (Lian et al., 2014, 2015). In 2017, Tianqi Capsule and Jinlida Granule were first included by the Chinese Diabetes Society in *Guidelines for the prevention and treatment of type 2 diabetes in China*. Ginseng is an important ingredient in Tianqi Capsule and Jinlida Granule. However, owing to the complex composition of Chinese herbal medicine, the mechanism of action is not clear. This has become a factor limiting the development of TCMs. Since Tu You You invented artemisinin to treat malaria and won the Nobel Prize, the use of Chinese medicine monomers in the treatment of disease has received widespread attention. Ginsenosides are the most important components of ginseng. Through review the articles, the mechanism of ginsenosides in treating diabetes is worth studying.

Ginsenosides has multiple targets to treat diabetes and diabetic complications *in vitro* and *in vivo* tests. Firstly, ginsenosides have anti-diabetic effects including insulinotropic action, reducing gluconeogenesis, improving insulin resistance, promoting glucose transport, regulating glucose tolerance and protecting islet cell to decrease blood glucose. Secondly, ginsenosides also have anti-inflammatory, myocardial protective, lipid-regulating, antioxidant, anti-angiopathic, immunosuppressive, anti-endothelial dysfunction and anti-neurotoxic effects. These effects could treat diabetic complications including diabetic nephropathy, diabetic cognitive dysfunction, diabetic cerebral infarction, diabetic cardiovascular complications and diabetic erectile dysfunction. Besides, researches have shown that ginsenosides have different therapeutic effects between T1DM and T2DM (Table 1). The role of ginsenosides in T2DM focuses on improving insulin resistance and treat T2DM through multiple targets, while ginsenosides have shown potential to participate in islet transplantation in T1DM.

**Table 1:** The difference between T1DM and T2DM.

	**T1DM**	**T2DM**
Compounds	Rh2; compound K; Re	Rg1; Rb3; compound K; Rg3; Rk1; Rg5; Rb1; Re; ginseng total saponins; malonyl ginsenosides; protopanaxadiol (PPD) and protopanaxatriol (PPT)-type saponins; Rb2; Re; Rd and R-Rh2
Target	PPARδ-STAT3 signaling; CD4(+), CD8(+) T cells; interleukin-2; interferon-γ; transforming growth factor-β; Foxp3; nuclear factor-κB, p38 MAPK, ERK1/2 and JNK signaling	FoxO1; AMPK; HNF4α; PGC-1α; STAT5-PPAR gamma pathway; endoplasmic reticulum stress; 11β-HSD1; JNK pathway; PI3K/Akt; mitochondrial function; GLP-1; glucose homeostasis; glucose disposal; Sirt1; autophagy; TNF-α; IL-6; SOD; MDA, phosphoenolpyruvate carboxykinase; glucose-6-phosphatase; microsomal TG transfer protein; 3-methylguanine; N2; N2-dimethylguanosine; acetoacetic acid; dodecanedioic acid; glycocholic acid; trehalose 6-phosphate; p38 MAPK; ERK1/2; cell viability and insulin signaling
Effects	Improving cardiac function and fibrosis; immunosuppressive effect and anti-angiopathy effects	suppressing of gluconeogenesis; improving insulin resistance; increasing insulin sensitivity; stimulating GLP-1 secretion; alleviating hyperglycemia; hepatoprotection; anti-apoptosis; attenuating diabetic myocardial damage; reducing inflammatory responses; regulating hepatic metabolism and lipid metabolism; regulating acid metabolism, energy metabolism, and gut flora metabolism; anti-angiopathy effects and protective astrocytes

Among the various ginsenosides, Rg1, Rg3, Rb1, and Compound K have the most promising therapeutic prospects for development as an adjuvant medicine for the treatment of diabetes. Rg1 has the potential to treat T2DM, T2DM with fatty liver disease, diabetic cerebral infarction, and diabetic cardiomyopathy complications. Experiments showed that Rg3 reduced blood glucose and increased plasma GLP-1 and plasma insulin through the improvement of insulin resistance, lipid metabolism, energy metabolism, and gut flora metabolism. A number of studies have shown that Rb1 may have some therapeutic effect on diabetic obesity as Rb1 decreased glucose tolerance, but increased insulin sensitivity, glucose consumption, and GLP1 secretion through the modulation of obesity-induced inflammation, central leptin sensitivity, and intestinal absorption. In a model of T2DM, compound K improved glucose intolerance, stimulated insulin secretion and insulin sensitivity through the suppression of hepatic gluconeogenesis and oxidative stress. In a model of T1DM, compound K exerted immunosuppressive effects that promoted islet transplantation.

Although ginsenosides have a wide range of antidiabetic effects for the prevention and treatment of DM and its complications, their use has some problems. A clinical trial reported that oral ginsenoside Re therapy failed to ameliorate beta-cell function or insulin sensitivity in diabetics with impaired glucose tolerance or in newly diagnosed overweight/obese subjects. Ginsenoside Re was not detected in human plasma after treatment (Reeds et al., 2011). This may be related to low bioavailability. The bioavailability of ginsenosides in the human body needs to be improved. However, the metabolic regulation effects of other ginsenosides have not been excluded. A research reported that Rg1 nanoparticle (PHRO, fabricated with γ-PGA, L-PAE (H), Rg1, and OX26 antibody) released Rg1 with sustained release manner and could promote the migration of cerebrovascular endothelial cells and tube formation and even penetrated the blood-brain barrier with high concentration in treating diabetic cerebral infarction (Shen et al., 2017). Nanotechnology may become a new tool to increase the bioavailability of ginsenosides. Current aptamer-based drug delivery technology is being developed for therapeutic use (Delac et al., 2015). So this might be a good method to provide a targeting tool for direct ginsenoside-loaded nanoparticle therapy. As we reviewed above, this article is limited to the study of mechanisms *in vivo* and *in vitro* experiments because of the lack of clinical trials. Nevertheless, ginsenosides could be considered for future development as a multi-target agent for therapeutic application of diabetes and its complications and more clinical trials are needed.

## Author contributions

LB and JG designed the work of review; LB, JG, and FW reviewed the literature available on this topic and wrote the paper; JZ and DW contributed in the scientific writing of the manuscript; LB, JG, and JW revised the manuscript. All authors approved the paper for publication. LB, JG, FW, JZ, and DW contributed equally to this work. LB and JG contributed equally to this study and share first authorship.

### Conflict of interest statement

The authors declare that the research was conducted in the absence of any commercial or financial relationships that could be construed as a potential conflict of interest.

## Supplementary material

Supplementary Table 1 *In vitro* and *in vivo* Tests of Ginsenoside in the Treatment of DM.

## Abbreviations

Ach: acetylcholine
AChE: acetylcholinesterase
AMP: activated protein kinase
BUN: blood urea nitrogen
JNK: c-Jun NH2-terminal kinase
DN: Diabetic nephropathy
DM: diabetes mellitus
FBG: fasting blood glucose
FOXO1: Forkhead transcription factor 1
GLP-1: lucagon-like peptide-1
CHOP: homologous protein
IDF: International Diabetes Federation
LDL-C: low-density lipoprotein cholesterol
MDA: malondialdehyde
MGR: Malonyl ginsenosides
NAFLD: Non-alcoholic fatty liver disease
Scr: serum creatinine
SGLT1: sodium-glucose cotransporters 1
SOD: superoxide dismutase
TC: total cholesterol
TG: triglyceride
T1DM: type 1 diabetes mellitus
T2DM: type 2 diabetes mellitus
TNF-α: tumor necrosis factor-alpha
TNF-β: tumor necrosis factor-β
IL-2: interleukin-2
IL-6: interleukin-6
PPAR-γ: peroxisome proliferator-activated receptor gamma coactivator 1-alpha.
